# Social Relations, Stress, and Racial Health Disparities

**DOI:** 10.1093/geroni/igab046.2242

**Published:** 2021-12-17

**Authors:** Kristine Ajrouch, Noah Webster, Toni Antonucci

## Abstract

This symposium brings together four papers that address racial health disparities by investigating stressful aspects of social relations at different points in the life course. Cleary and colleagues focus on racial disparities in psychological health by testing cross-sectional effects of intergenerational stress over time. In particular, they investigate effects of network composition on the relationship between mothers' stressors and their children's depressive symptoms at three time points over 23 years. Camacho and colleagues use longitudinal data from the National Social Life, Health and Aging Project to examine cognitive decline among U.S. African-American, Latino, and White adults aged 60 and above. Results indicate loneliness predicted greater global cognitive decline over time in all groups. However, race differences in this association were found across cognitive function domains. Turner and colleagues consider dementia caregiving challenges among non-Hispanic Blacks. Data from five focus groups were analyzed to reveal distinctive challenges to caregiver health during the COVID-19 pandemic including increased burden and barriers to service access. Finally, Sol and colleagues examined the bidirectional association between loneliness and self-rated health over time among a racially diverse sample. Findings illustrate racial patterns in how loneliness at midlife influences health in later life. Antonucci will discuss the role of stress from social relations as a means to fully understand racial disparities in health across the life course.

